# Supplementary material for: The early local and systemic Type I interferon responses to ultraviolet B light exposure are cGAS dependent
Source: Sci Rep. 2020 May 13;10:7908. doi: 10.1038/s41598-020-64865-w (PMC7220927; doi:10.1038/s41598-020-64865-w)
Supplement: Supplementary file 1 — Supplementary information. [file 41598_2020_64865_MOESM1_ESM.pdf]

The early local and systemic Type I interferon responses to ultraviolet B light exposure are cGAS dependent.

Authors:

Sladjana Skopelja-Gardner, Jie An, Joyce Tai, Lena Tanaka, Xizhang Sun, Payton Hermanson, Rebecca Baum, Masaoki Kawasumi, Richard Green, Michael Gale, Jr., Andrea Kalus, Victoria P. Werth, Keith B. Elkon

SUPPLEMENTARY INFORMATION

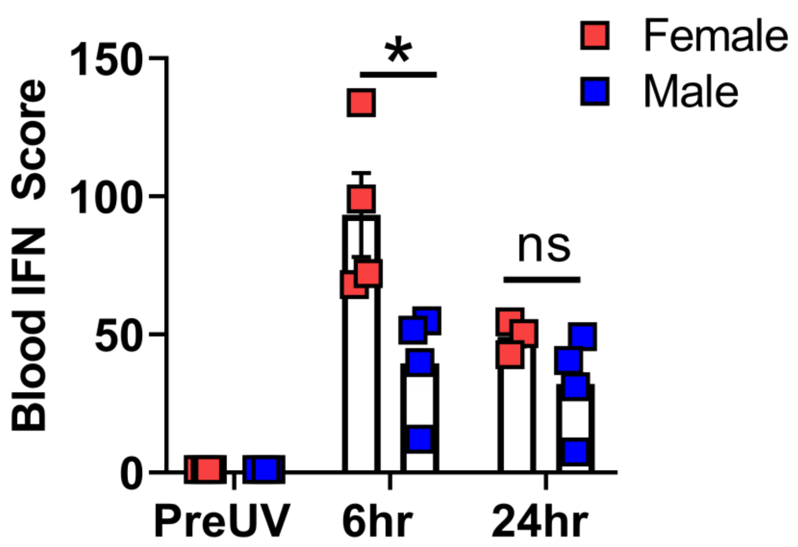

Supplementary Figure S1. Early blood interferon response is higher in female compared to male B6 mice following exposure to UVB light. Female and male age-matched B6 mice were exposed to a single dose of UVB light as in Fig. 1. Blood IFN scores were calculated as the sum of normalized relative expression levels of the 7 most highly expressed ISGs after UV exposure (*Mx1, Ifit1, Ifit3, Ifi44, Usp18, Oasl1,* and *Ifi27l2a)*. Statistical significance was determined by Student's t-test (n=4 per sex, *p<0.05, ns = not significant).

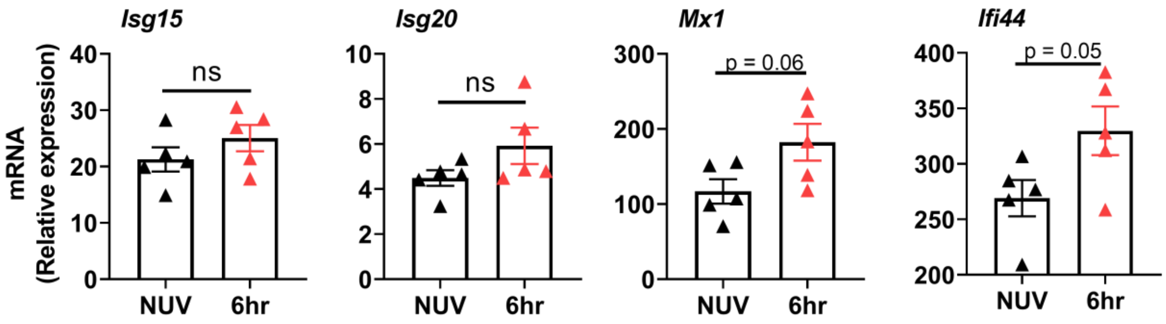

Supplementary Figure S2. Interferon Stimulated Gene (ISG) expression in the kidney early after skin exposure to UVB light. Relative expression of representative ISG transcripts in the perfused kidney tissues of B6 mice at baseline (no UV, NUV) or 6h after skin exposure to UVB light. Statistical analysis was performed by Student's t-test (n=5 per time point, ns = not significant).

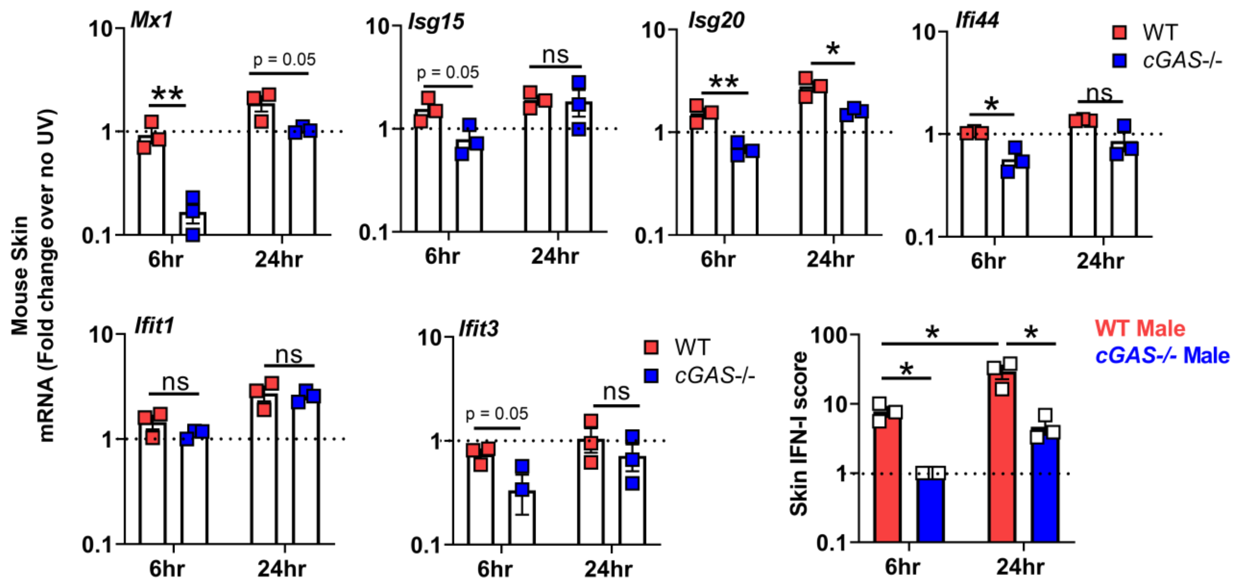

Supplementary Figure S3. Type I interferon (IFN-I) response in the skin after exposure to UVB light in B6 and *cGAS-/-* male mice. Age-matched male B6 (wild type, WT) and *cGAS-/-* mice were exposed to a single dose of UVB light as in Fig. 1. Skin biopsies were obtained prior to UVB light exposure and at 6 and 24h after irradiation. Fold change in the expression of IFN-I stimulated genes (ISG) in the skin was determined relative to baseline, i.e. non-irradiated skin. Skin IFN scores at 6 and 24 hours after UVB were calculated as sum of normalized expression levels of the same 6 ISG. Statistical significance was determined by Student's t-test (n=3 per genotype per time point; *p<0.05, **p<0.01, ns = not significant).

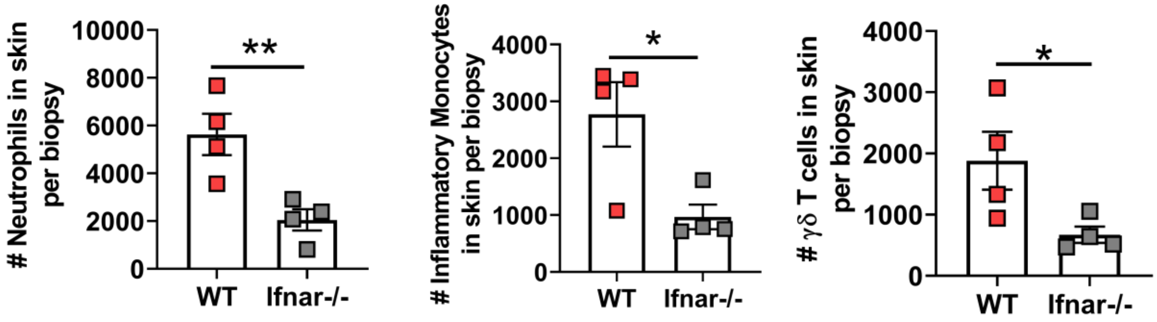

Supplementary Figure S4. IFNAR deficiency results in decreased cellular inflammation following skin exposure to UVB light. Age-matched B6 (wild type, WT) and *Ifnar-/-* mice were exposed to a single dose of UVB light as in Fig. 1. Flow cytometry analysis of skin was performed and the number of neutrophils (CD45+CD11b+Ly6C^{int}Ly6G^{hi}), inflammatory monocytes (CD45+CD11b+Ly6C^{hi}Ly6G^{neg}), and γδ+ T cells (CD45+CD11b- γδ^{+}) determined based on total cell number per skin biopsy (6 mm). Statistical significance was determined by Student's t-test (*p<0.05, **p<0.01).

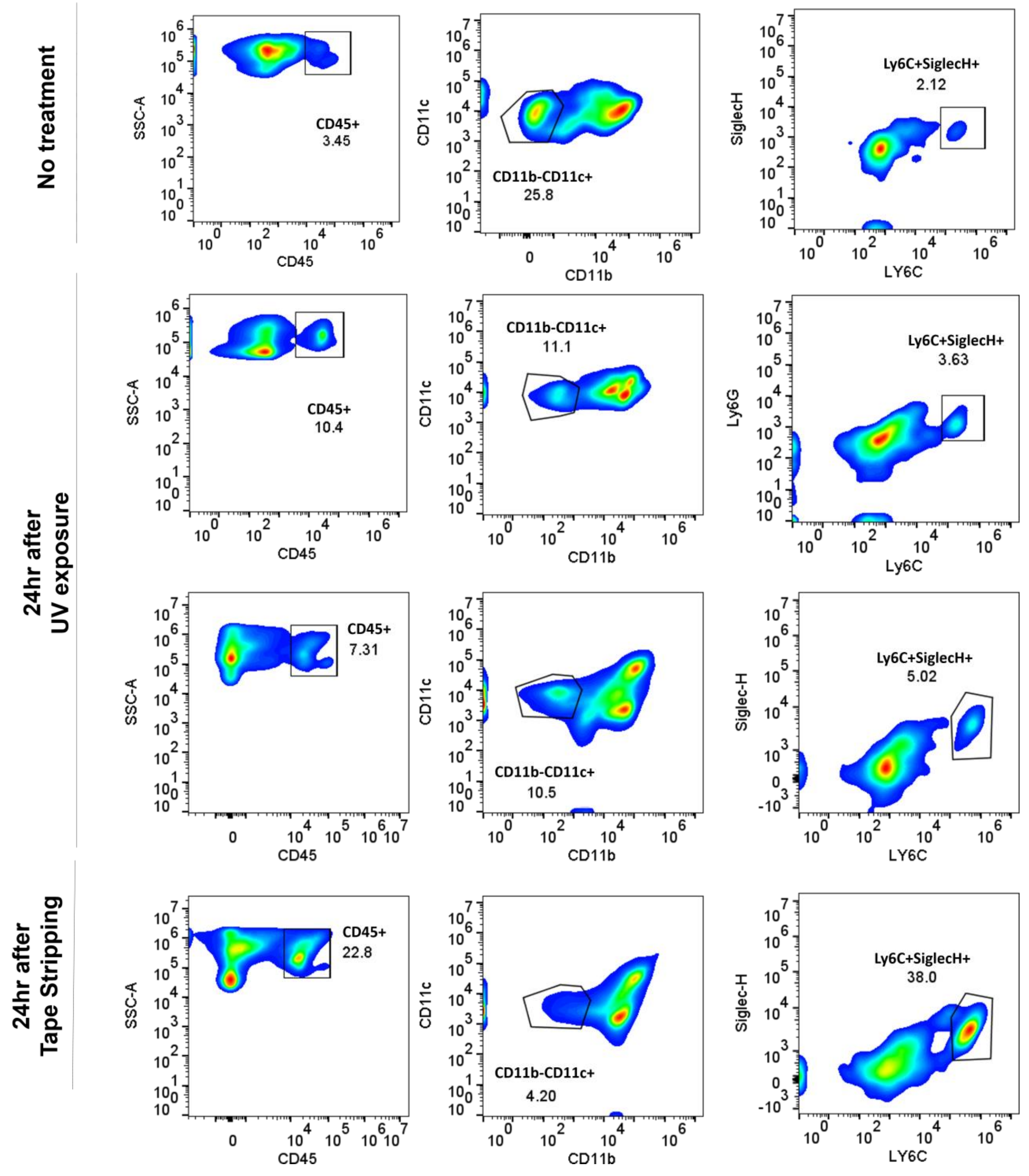

Supplementary Figure S5. Plasmacytoid Dendritic Cell (pDC) detection the skin. Flow cytometry was performed to analyze pDC presence in untreated skin (top panel), skin 24hr after exposure to a single dose of UVB light (middle 2 panels), and in the tape-stripped skin as a positive control (bottom panel). PDC were detected by SIglecH expression in CD11b-CD11c+ immune cell (CD45+) population.

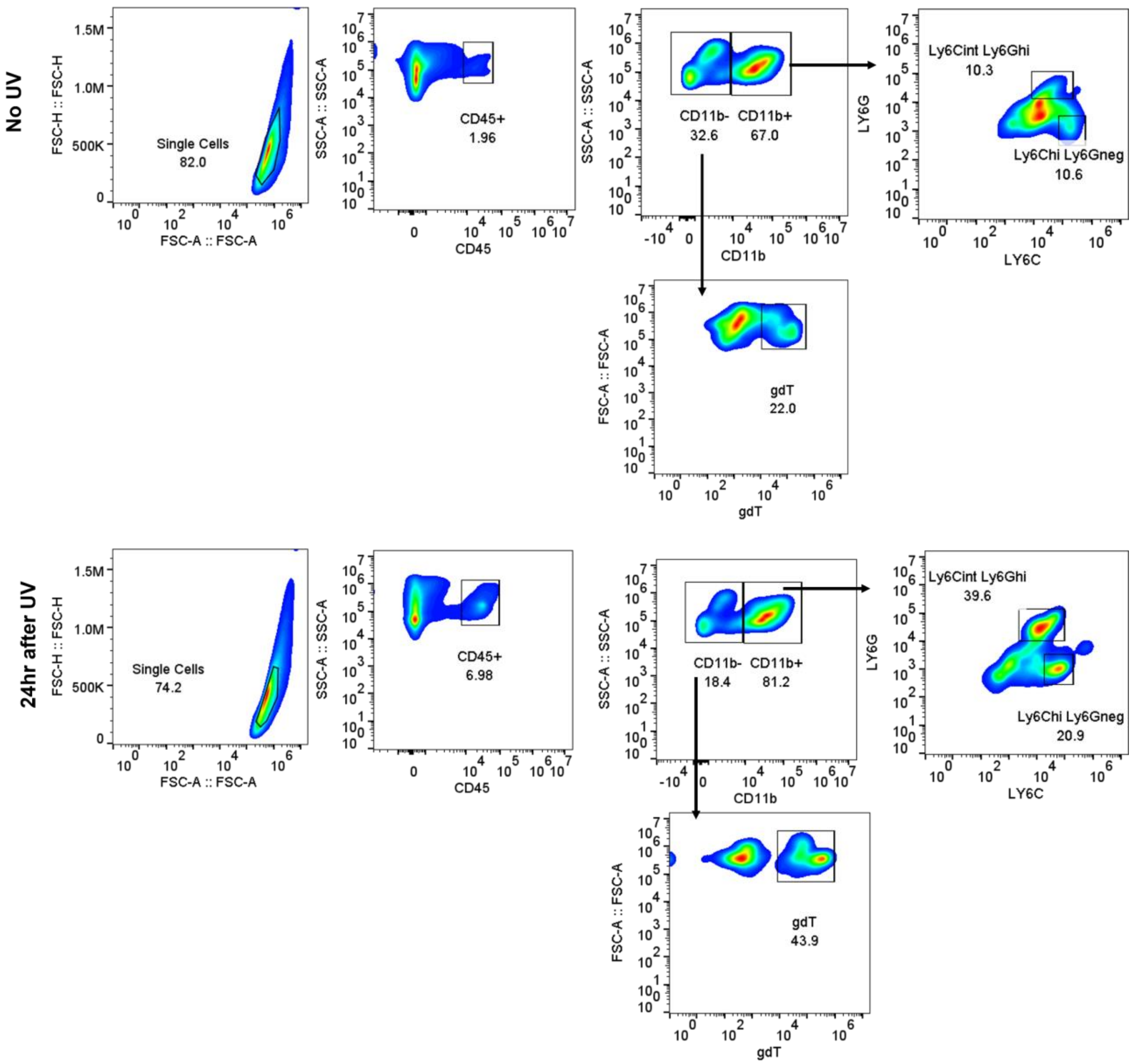

Supplementary Figure S6. Flow cytometry gating of innate immune cell populations without UV exposure (No UV, top panels) and 24hr after UV exposure (bottom panels). Immune cells (CD45+) were gated on within the single cell populations. Neutrophils (Ly6CintLy6Ghi) and inflammatory monocytes (Ly6ChiLy6Gneg) cells were gated on within CD45+CD11b+ cells. Γδ T cells were gated on within CD45+CD11b- cell populations.

Supplementary Table S1. Primer Sequences

Gene	Forward	Reverse	BLAST match accession no.
18s	AACTTTCGATGGTAGTCGCCGT	TCCTTGGATGTGGTAGCCGTTT	NR_003278.3
Gapdh	TGGAAAGCTGTGGCGTGAT	TGCTTCACCACCTTCTTGAT	NG_007785.2
Isg15	AAGCAGCCAGAAGCAGACTC	CACCAATCTTCTGGGCAATC	NM_015783.3
Isg20	TCACGGACTACAGAACCCAAG	TATCCTCCTTCAGGGCATTG	NM_001291221.1
Irf7	GTCTCGGCTTGTGCTTGTCT	CCAGGTCCATGAGGAAGTGT	NM_016850.3
Mx1	CCTCAGGCTAGATGGCAAG	GGCAGACACCACATACAACC	NM_010846.1
Ifit1	TGCTGAGATGGACTGTGAGG	CTCCACTTTCAGAGCCTTCG	NM_053217.3
Ifit3	TGGCCTACATAAAGCACCTAGATGG	CGCAAACTTTTGGCAAACTTGTCT	NM_010501.2
Ifi44	AACTGACTGCTCGCAATAATGT	GTAACACAGCAATGCCTCTTGT	NM_133871.3
Usp18	TTGGGCTCCTGAGGAAACC	CGATGTTGTGTAAACCAACCAGA	NM_011909.2
Oasl1	CAGGAGCTGTACGGCTTCC	CCTACCTTGAGTACCTTGAGCAC	NM_001359945.1
Ifi27l2a	CTGTTTGGCTCTGCCATAGGAG	CCTAGGATGGCATTTGTTGATGTGG	NM_029803.3
Il6	TCTATACCACTTCACAAGTCGGA	GAATTGCCATTGCACAACTCTTT	NM_031168.2
Tnf	CTGAACTTCGGGGTGATCGG	GGCTTGTCACTCGAATTTTGAGA	NM_013693.3
IL1b	TCCAGGATGAGGACATGAGCAC	GAACGTCACACACCAGCAGGTTA	NM_008361.4
Cxcl1	CAATGAGCTGCGCTGTCAGTG	CTTGGGGACACCTTTTAGCATC	NM_008176.3
Ccl2	CCCAATGAGTAGGCTGGAGA	AAAATGGATCCACACCTTGC	NM_011333.3
Cxcl10	GACGGTCCGCTGCAACTG	CTTCCCTATGGCCCTCATTCT	NM_021274.2
